# Bread wheat: a role model for plant domestication and breeding

**DOI:** 10.1186/s41065-019-0093-9

**Published:** 2019-05-29

**Authors:** Eduardo Venske, Railson Schreinert dos Santos, Carlos Busanello, Perry Gustafson, Antonio Costa de Oliveira

**Affiliations:** 1 Plant Genomics and Breeding Center, Crop Science Department, Eliseu Maciel College of Agronomy, Federal University of Pelotas, Capão do Leão Campus, Capão do Leão, Rio Grande do Sul 96010-610 Brazil; 2 Plant Sciences Division, 1–32 Agriculture, University of Missouri, Columbia, MO 65211 USA

**Keywords:** Hexaploid wheat, Agriculture, Genetic resources, Biotechnology, Genomics

## Abstract

**Background:**

Bread wheat is one of the most important crops in the world. Its domestication coincides with the beginning of agriculture and since then, it has been constantly under selection by humans. Its breeding has followed millennia of cultivation, sometimes with unintended selection on adaptive traits, and later by applying intentional but empirical selective pressures. For more than one century, wheat breeding has been based on science, and has been constantly evolving due to on farm agronomy and breeding program improvements. The aim of this work is to briefly review wheat breeding, with emphasis on the current advances.

**Discussion:**

Improving yield potential, resistance/tolerance to biotic and abiotic stresses, and baking quality, have been priorities for breeding this cereal, however, new objectives are arising, such as biofortification enhancement. The narrow genetic diversity and complexity of its genome have hampered the breeding progress and the application of biotechnology. Old approaches, such as the introgression from relative species, mutagenesis, and hybrid breeding are strongly reappearing, motivated by an accumulation of knowledge and new technologies. A revolution has taken place regarding the use of molecular markers whereby thousands of plants can be routinely genotyped for thousands of loci. After 13 years, the wheat reference genome sequence and annotation has finally been completed, and is currently available to the scientific community. Transgenics, an unusual approach for wheat improvement, still represents a potential tool, however it is being replaced by gene editing, whose technology along with genomic selection, speed breeding, and high-throughput phenotyping make up the most recent frontiers for future wheat improvement.

**Final consideration:**

Agriculture and plant breeding are constantly evolving, wheat has played a major role in these processes and will continue through decades to come.

## Background

Bread wheat (*Triticum aestivum* L.) is one of the most important crop species, responsible for the emergence and development of agriculture and has fed, and continues to feed, a large part of the world’s population across many centuries [97, 106]. Wheat has been improved by man over the last 8000 to 10,000 years ago when the species first arose. Initially it happened in an unconscious way, then intentionally, but empirically, and then, for more than a century, based on scientific knowledge [18, 64].

Wheat breeding, as for many other crops, has been evolving fast, both in terms of basic science, methods and tools. The literature on wheat breeding is vast, including countless scientific papers, reviews and even dense book collections already published. Therefore, all relevant aspects and examples cannot be covered in a single text. On the contrary, we do encourage readers to go through this bibliographic ever growing wealth for a deeper understanding on any given topic. Thus, the objective of this review is to provide a brief and valuable synthesis on some selected aspects related to the history, but especially, current advances in wheat breeding, devoted especially to students and researchers with little or even no knowledge on the theme. Through this review, the reader can have a quick and general overview on the discussed topics and, when necessary, get a direction to start searching for further literature, as we have tried to cite the most important and recent papers on each topic. Therefore, in the next sections we show the origin of this species and how it became so important with a brief history of wheat cultivation and breeding. Priorities and particularities of wheat breeding are presented. Special consideration is given to new approaches and tools that are currently under development, and the ones that lately reappeared. Finally, the promising future and perspectives are discussed.

### Origin and importance

#### One of the fathers and lifelong ally of agriculture

Bread or common wheat is undoubtedly one of the most important cultivated plants, in fact, in addition to its ancestry, the cereal represents a large part of the history of agriculture itself [8, 18, 44, 58, 93, 97].

Today, wheat is the basis of a significant part of the world’s diet, being an important source of energy (providing ca. of 20% of world population demand), and protein (also providing ca. 20%), as well as vitamins and other beneficial compounds, not only for humans, but also as animal feed [42, 106].

It is grown from 67° North to 45° South, including a wide range of altitudes, but it is less cultivated in tropical regions [33]. In 2016, more than 749 million tonnes of this cereal were produced on 220 million hectares around the world, which puts wheat in second place regarding production among the cereal crops (behind maize - *Zea mays* L.) but in the first position regarding area harvested amongst all crops [32]. Approximately 95% of wheat cultivated is hexaploid with the remaining 5% being durum wheat (*T. turgidium* L.) and few other less important types [106].

#### The origin of the species

Bread wheat is an allohexaploid species (2*n* = 6× = 42, AABBDD genomes), resulting from the combination of 3 interrelated diploid genomes [28, 66, 79, 83]. Donors of the A genome (*T. urartu*) and B genome (closely related to *Aegilops speltoides*), diverged from a common ancestor about 7 million years ago. These two species first generated (~ 5.5 million years ago) the donor of the D genome (*Ae. tauschii*), through hybridization and homoploid speciation. Less than one million years ago emmer wheat (*T. turgidum*), an allotetraploid with AABB genomes became into existance. Finally, from 8000 to 10,000 years ago, probably in the Fertile Crescent, in a region that nowadays comprises Northern Iran, the hybridization between *T. turgidum* and *Ae. tauschii* gave rise to the hexaploid *T. aestivum*, which after domestication and centuries of cultivation and selection, resulted in the bread wheat that is cultivated today [27, 28, 53, 67, 68, 79, 83, 98].

Unlike other cultivated species, hexaploid wheat was not selected from a wild species, but arose from the hybridization between a species already cultivated by man that time (emmer wheat), so it is possible to say that maybe there was never any *T. aestivum* in the wild [106]. The reasons why this cereal became so widely adopted by man include its high environmental adaptability, thanks to its allopoliploid nature, which has conferred to wheat the so-called “genomic plasticity”. Also, due to its excellent food/feed qualities, not only regarding carbohydrates, proteins and vitamin content, but also for the unique elastic property of its gluten, which provided a more diverse use for its flour [27, 106].

#### The beginning and evolution of wheat cultivation and breeding

The emergence of modern *T. aestivum* occurred due to agriculture. Thanks to growing its ancestor (emmer) in an area with spontaneous occurrence of *Ae. tauschii*, the inter-specific hybridization that generated this species occurred [27]. After its emergence, cultivation gradually began to predominate around its center of origin and then expanded to several regions of the globe, improved by natural selection and man in an unintentional way [18].

The “intentional” breeding, even if empirical, began at the end of the XVIII century. The first reported attempts to allow for cross-fertilization of different types of plants was made by Knight (1787) in England. These crosses allowed for the observation of improvements especially for disease resistance [64]. At the end of the XIX century, Vilmorin, in France, and Rimpau in Germany, amongst other breeders, made important contributions in the development of superior wheat genotypes by man-made hybridization or simply selection, motivated by Darwin [22, 23], but occurred without a clear understanding of important foundations of their work [64]. Breeding from a solid scientific base began only after the rediscovery of Mendel’s findings, at the beginning of the last century. Biffen’s classic work [7] was probably the first to validate such knowledge in wheat, once again focusing on disease resistance. Nilsson-Ehle [76] greatly contributed to the study of quantitative traits involving grain color in wheat.

Other advances took place gradually over the decades, until a major leap was made with the so-called “Green Revolution” of the mid-1960s, when wheat and rice (*Oryza sativa* L.) were protagonists [9, 29, 80, 91]. This revolution consisted in the development of “modern” cultivars - those of wheat mainly by CIMMYT, the International Center for Maize and Wheat Improvement, Mexico. Those were short statured (semi-dwarf), photoperiod insensitive and high yielding spring cultivars. This was only possible due to the incorporation of the genes *Reduced height* (*Rht*) and *Photoperiod* (*Ppd*), which have had extremely important effects on the adaptability of this species. *Ppd-D1a,* which is an insensitive allele to the photoperiod that reduces flowering time, and *Rht-B1b* and *Rht-D1b*, which makes the cereal insensitive to gibberellin, shortened plant’s stature. These genes are today widespread in the wheat elite germplasm all around the world and new alleles are still under study, with potential to contribute to this trait [10, 125, 128].

These new genotypes became widely adopted, especially in developing countries, and generated an impact on the reduction of hunger and poverty, with huge repercussions [9, 29, 78, 80]. The Nobel Peace Prize awarded to Dr. Norman E. Borlaug deserves a special mention here, due to his decisive role in this revolution [9].

**Fig. 1:**
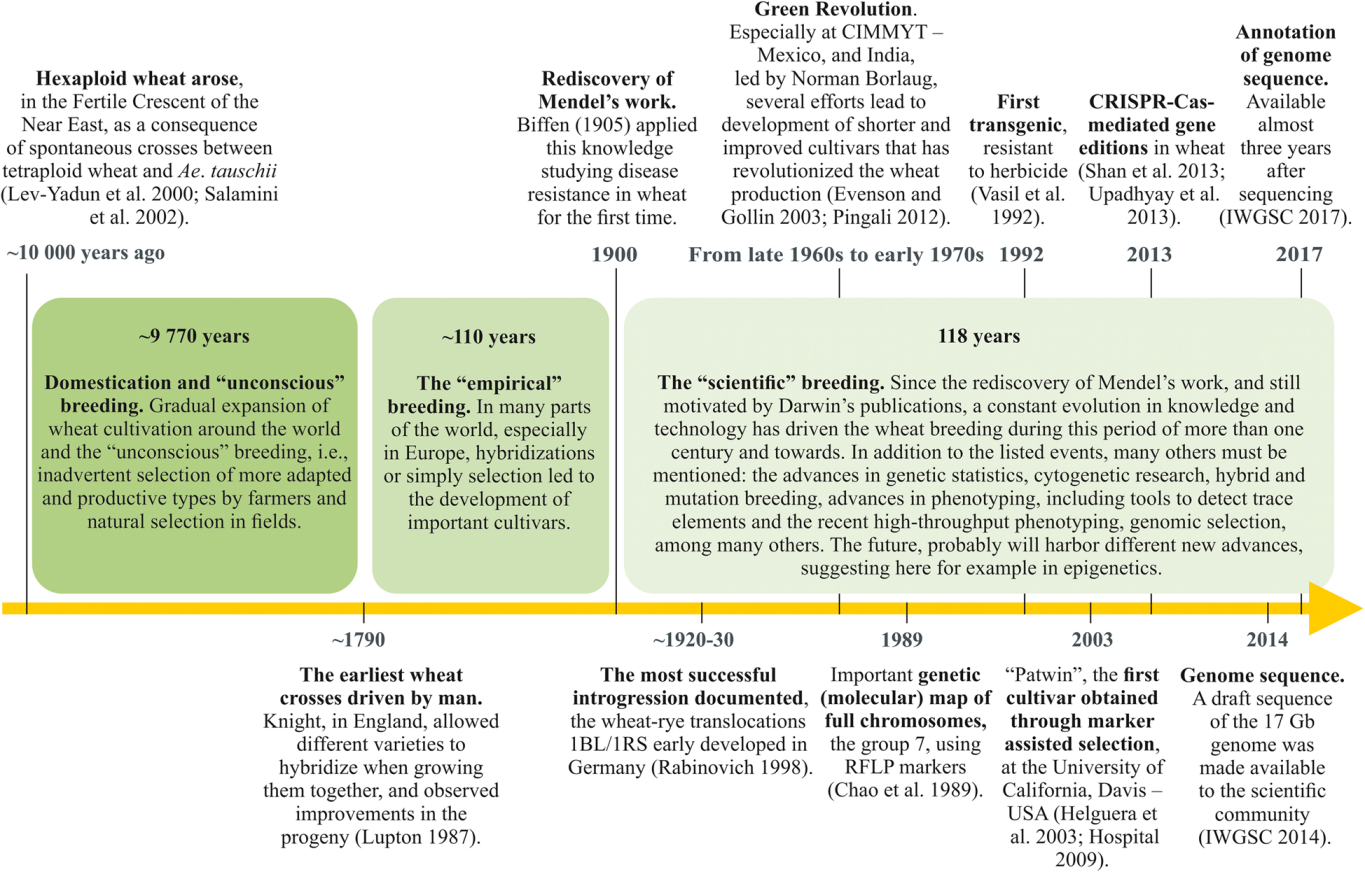
Wheat breeding timeline. Three main phases can be defined in wheat breeding history: the “unconscious”, the “empirical” and the “scientific” breeding, this latter is illustrated with several important events

Since then, wheat breeding has advanced even further with new technologies such as molecular markers, the recent availability of a reference genome sequence and annotation, and even the recent use of techniques such as genome editing, genomic selection, speed breeding and high-throughput phenotyping. The evolution of wheat breeding accross time is briefly illustrated in Fig. 1, highlighting phases and important events.

### Wheat breeding: priorities and some general aspects

#### The priorities in wheat breeding

The main objectives of wheat breeding have been similar over many decades. Increasing the yield potential has been prioritized in order to meet the food requirements of an ever increasing population [9, 80].

Probably the second most important trait is disease resistance, as from the first breeding attempts by Knight in 1787 until today, in different countries [64]. For instance, “old diseases”, such as the rusts, are still a cause of concern for wheat cultivation, but new ones are appearing, such as wheat blast, considered one of the most recent and concerning threat for wheat cultivation worldwide [127].

Third, is tolerance to abiotic stresses, especially drought and heat – the latter is a borderline to cereal crop expansion, cold and acid soils (aluminum), and various quality traits. Finally, all the others must come, such as resistance to insects, lodging, double-purpose (forage and grain), and improved nutrient use and grain biofortification efficiency, among numerous others. This ranking is based on a general overview on the vast available literature, however this order of priority more than certainly varies within each environmental region and over time.

As already mentioned, publications on wheat breeding are vast, fortunately there has been a number of reviews already published, which summarize the most important steps already taken for different traits, ie., yield potential [29, 91], stem rust resistance [107], drought tolerance [74] and biofortification, which should grow in importance over the next few years [129].

#### New priorities in wheat breeding

Most future priorities in wheat breeding should remain the same, but the need for faster development and accumulation of knowledge from different fields should provide new strategies and paths to reach these goals. Increasing photosynthetic capacity has been shown to be one of the most important barriers to improve wheat yield potential and there is theoretical evidence that it could be enhanced by the insertion of genes for C4 carbon fixation, whose strategy has merited investment [87, 90].

Wheat grain is known to be rich in gluten, a trait that is critical for baking, but negative for consumption by celiac, and also non-celiac gluten-sensitive people has been a largely discussed topic among nutritionists [15, 37]. This may lead to a potential reduction in wheat consumption in the coming decades, unless we can provide grain that does not possess this disadvantage. Fortunately, there is evidence of some wheats that possess a gluten, but of a chemically different type, which can be consumed by people with celiac disease, potentially becoming an important target for wheat breeding in forthcoming years [95, 111].

#### Special aspects on wheat breeding

Wheat is a self-pollinated species. Therefore, the conventional structure of its breeding programs do not differ much from other autogamous plants. It includes the use of artificial hybridizations between previously selected genotypes, something already performed for more than two centuries, and different forms of selection within segregating populations [64, 100]. It is recognized that these processes were, and will continue to be, the main responsiblity for the development of wheat cultivars worldwide. However, new tools and approaches are assisting this process, increasing its success rate and diminishing costs, time and labour.

Improving wheat may be more difficult than for many other crops, since the breeder needs to “match” quantity and quality, allying yield with grain and flour quality, which needs are not a constant concern for crops like soybean (*Glycine max* L.) or maize (*Zea mays* L.), which can, for the most part, focus on yield [106]. Also, it is a species with restricted genetic variability when compared to most of other crops. Moreover, its genome size, complexity and polyploid nature constitute a challenge when applying some biotechnological techniques.

#### The restricted genetic diversity

Wheat is recognized to have restricted genetic variability, when compared to most other crops [18, 20]. This is due to several reasons: 1) it is an allohexaploid generated by crosses involving three highly interrelated diploid species, and poplyploidization is a force which restricts itself genetic variability; 2) another reason, suggests that few plants of the ancestral species were involved in the formation of wheat, also restricting its initial genetic variability [27, 58]; 3) Finally, it is a young species, ca. 8000 to 10,000 years old, which is insufficient time for the species to accumulate mutations or to receive genes or alleles by natural or artificial interspecific cross-breeding processes [20, 28, 66].

Domestication, centuries of cultivation, and modern breeding have further restricted the genetic variability of several cultivated species, and wheat is among them [34, 71, 89, 119]. It is important to remember that wheat was one of the first species to be domesticated and cultivated, further decreasing its variability due to constant selection cycles since then [18, 58, 93]. The impact of the narrowing of wheat variability is visible through current projections, which show that the cereal might not meet its demand in few decades [88], unless measures are taken in order to broaden its genetic base.

To broaden the genetic diversity available for wheat breeding, different techniques will need to be applied, including induce mutation, genetic transformation, genome editing, and introgressions from species of the secondary and tertiary gene pools.

### Resurgent and current approaches in wheat breeding

#### Introgressions

Among all crop species, wheat is probably the one in which most research has been invested regarding the use of wild and cultivated relatives as source of variability for its improvement. The attempt to incorporate traits of related species into wheat germplasm is not new. In fact, the attempts in this sense began long ago, as early as plant breeding itself [6]. If, on one hand, wheat is restricted in variability within its germplasm, there is an immeasurable richness in variation found in related species belonging to its secondary and tertiary gene pools [25, 102, 131].

The most important introgression to date in wheat involved a chromosomal translocation *1RS-1BL* between wheat and rye (*Secale cereale* L.), generated in the first third of the last century, which increased wheat yield potential and resistance/tolerance to biotic and abiotic stresses. This segment is still present in many of important cultivars currently used [21, 85, 101]. The researcher E.R. Sears deserves also a special mention here, due to his great contribution to this field. Today, there are several excellent chromosome manipulation studies in progress (e.g. [54]). However, there is a consensus that the practical use of introgressed genes in the development of superior cultivars has in the past been very limited and should be further explored [132].

Another strategy in this field is the development of synthetic wheat, repeating the interspecific crosses that occurred in nature that led to the formation of hexaploid wheat [61, 130]. In this method, different accessions of the species *T. monococcum*, *T. turgidum,* and *Ae. tauschii* can be used for the formation of new genetic constitutions of wheat, greatly increasing the genetic variability of the primary gene pool [73]. Numerous synthetic wheat germplasm pools have been developed by CIMMYT [130]. This illustrates an advantage that wheat possesses, as an allohexaploid, when compared to diploid species.

The use of other species in wheat pre-breeding programs has been an important field of research (for a complete review, see [72]). Recently, however, it seems to be reaching a new momentum, driven by a remarkable shortage of genetic diversity in wheat, accompanied by an increased need for improved adaptability for the crop. This adaptability is needed to counteract the unfavorable conditions brought by the ongoing climate changes. Enhanced technologies for introgression detection, such as high-throughput genotyping, have motivated investiments in this field. Other potential approaches, such as gene editing will be further discussed in a dedicated section [12, 54, 131].

#### Mutagenesis

Mutation induction, whether via chemical or physical mutagens, has been widely used in order to increase the genetic variability in several cultivated species, including wheat [77]. The polyploid nature of wheat confers a kind of buffer effect, in which mutations in one of its genomes can be compensated by homoeologous genes masking their effect making them difficult to be detected [77]. Fortunately, TILLING methods [108, 114] and high-resolution melting analysis [26] have proven to be efficient for the detection of mutations in the different genomes of hexaploid wheat.

From 1960 to 2017, 256 wheat cultivars were generated by mutagenesis in different countries and have been registered in the FAO/IAEA database (https://mvd.iaea.org). In this repository [31], all cultivars are described with information about how the mutations were induced and focuses on the value-added attributes. Among the many examples of agronomically important mutations are resistance to herbicides of the imidazolinones group [84] and increases in amylose content and starch resistance [109].

#### Molecular markers and new genotyping approaches

The use of molecular markers for QTL mapping and marker-assisted selection (MAS), such as for resistance to fusarium head blight [13] and drought [39] has been growing and the accumulation of data generated during the past decades has allowed us to perform different meta-analyses [39]. From the 1990s to 2000s, AFLP, RFLP, and SSR were the most used markers [17, 40, 46, 75, 110]. However, recently a revolution occurred, in which science changed from the use of a few markers, from the types mentioned above, to thousands of single nucleotide polymorphism (SNP) markers using high-throughput platforms. This was initiated with DArT markers [1] and then with SNPs evaluated through genotyping arrays such as *Illumina*® *9 K iSelect Beadchip Assay* [16], *Illumina*® *iSelect 90 K SNP Assay* [121] and *Axiom*® *820 K SNP array* [126], in which respectively 9000 to nearly 820,000 SNPs can be evaluated in a single analysis. Also, using genotyping by sequencing (GBS), thanks to the arrival of next generation sequencing technologies, maps containing 20 to 450 K loci have already been generated for wheat [82, 96].

Similarly to other crops, genetic mapping also evolved from mapping populations generated from crosses between only two contrasting parents to genome-wide association studies (GWAS), in which hundreds of diverse accesses are evaluated on each study, thus allowing the capture of a larger genetic diversity and a deeper look in the causal variation between agronomically interesting phenotypes [3, 14, 38, 56, 60, 81].

#### Genomic selection

Although Marker Assisted Selection (MAS) has proven to be useful in a number of situations in wheat breeding, it has the limitation of being only able to aid the selection for a few genes or alleles at a time. However, it is well known in crop breeding that most agronomic traits present a quantitative nature, are governed by numerous genes, most of these with very small effect on the phenotype. In this regard, genomic selection (GS) came as a revolutionizing ally, also in animal breeding [69]. The approach aims ultimately to perform selection and prediction of breeding values based only on genotyping, within a model calibrated with phenotypic values, and with a whole genome perspective, i.e., taking into account genomic polymorphisms in linkage disequilibrium with as many as possible genes with effect on a given trait [51].

The number of studies applying GS in wheat breeding are at an increasing rate. One of the main measures to assay the effectiveness of GS is its accuracy, i.e., how much the prediction compares with the real phenotypes. Applying genotyping by sequencing, GS for wheat yield under irrigated and drought conditions showed accuracies of 0.28 and 0.45, respectively, which are low to moderate values [81]. On the other hand, GS for fusarium head blight resistance showed moderate to high accuracies, being 0.82 the highest value found, for fusarium damaged kernels trait [4]. High accuracies are pursued in this approach, and many factors affect its value, such as the heritability of the trait, the number and quality of the markers, the GS statistical model adopted, among others [43]. In this regard, Bassi et al. [5] proposed different schemes dedicated to the implementation of GS in wheat breeding.

### The reference genome sequence and annotation

In 2005, efforts to generate a reference genome of wheat for the scientific community began, with the establishment of the International Wheat Genome Sequencing Consortium (IWGSC). Nine years latter, in 2014, the first version of this sequence, still considered as a draft, was published for the hexaploid wheat cultivar Chinese Spring [47]. This huge and complex sequence, estimated in 16 to 17 Gb in total, has been gradually assembled, improved and made available through the repository of the consortium (https://www.wheatgenome.org). Finally, after another 3 years, a first version of the annotation has been made available [48], which has also been continuosly improved [49]. In addition to IWGSC, another research group was responsible for the first near-complete assembly of the hexaploid bread wheat genome, with a total of 96% of its sequence, also of Chinese Spring [136].

Now these reference genomes, especialy the one made available by IWGSC, through its platform for public access, are a powerful tool for breeding and other genetic studies on this crop, being used to better understand wheat evolution [28, 66] and for genome wide association studies [3], among many other examples of use.

The completion of the first wheat reference genome of the Chinese Spring cultivar has been considered a step-change by researchers. However, it is obvious that more representatives from the species should also be sequenced, for a more effective use of genomics in breeding. It motivated the establishment of *10+ Wheat Genomes Project* \ (http://www.10wheatgenomes.com/). This global partnership aims to characterize the wheat ‘pan genome’, and will generate at high quality wheat genome assemblies and develop strategies and resources to compare multiple wheat genome sequences from around the world.

### Hybrid breeding

In some crops, such as maize and rice, the development and cultivation of hybrid cultivars is common, not recent and with clear advantages over the cultivation of open pollinated populations or inbred lines. For wheat, however, less than 1% of the area is cultivated with hybrids [52, 63]. After unsuccessful attempts during the past decades, research in the development and cultivation of hybrids seems to be becoming one priority in wheat breeding [63, 124].

This is due to a huge accumulation of knowledge and new technologies, and recent results are promising. The use of genomic tools to analyze the heterotic pattern among large groups of lines has proved to be efficient in obtaining highly productive hybrids [135], with genome wide selection being the most advantageous method of prediction [60]. In this sense, several hybrids have shown to be highly advantageous regarding yield [62] and resistant to diseases [70], while several difficulties associated with seed production are being overcome [124].

### Genetic transformation (transgenics)

The cultivation of transgenics is still a debate topic in our society. Its acceptance is not unanimous around the world, either because of social or religious reasons [106]. The scientific results have not been able to overcome the fear on its potential effects on human health [45, 65]. This is why there are not many records of the use of transgenic wheat cultivars [116], not allowing its comparison with crops such as soybean, maize or cotton, even after 27 years of the first transformed wheat [117]. Indeed, authors have termed wheat as the cereal abandoned by GM [127]. Research results, however, have been encouraging, generating genotypes with improved resistance to powdery mildew (*Blumeria graminis*) [134], leaf spot caused by *Bipolaris sorokiniana* [50] and fusarium head blight (caused mainly by *Fusarium graminearum*) [59]. Also, tolerance to drought [118], salinity and freezing [35] and even improvement in baking traits [86] have been achieved, among other traits [116]. Another alternative tool is the creation of cisgenic plants, where transferred genes come from the same species, something that has proven to be more easily accepted by society [113]. Despite these considerations, genetic transformation has been quickly replaced by genome editing, a very powefull approach, as presented in the next topic.

### Genome editing

Among the most recent and promising innovations in terms of biotechnology and plant breeding involves genome or gene editing [11, 99]. This technique can accurately target segments of the genome for modification, either by deletion, insertion or substitution of nucleotides [99]. In wheat, despite the great complexity of its extensive, redundant, and polyploid genome, several attempts have proven to be successful [105, 115, 122, 133]. Even a specific protocol for this species has already been established using the CRISPR/Cas9 system [104]. Among the most exciting results obtained with this technique is the simultaneous modification of three homoeo-alleles of the same gene, i.e., being capable of modifying this gene in all three different genomes, demonstrating the precision that these methods have been able to reach [122].

Gene editing can also be applied as a tool for gene introgression from wild relatives into wheat background, in which the linkage drag can be mitigated by precise gene replacement [120].

### Meiotic recombination manipulation

Crop breeding relies largely on meiotic recombination, which allows for recombination of genes/alleles in different new genetic compositions, thus allowing selecting new improved cultivars [57]. Controlling this process would be of high interest for breeders. In bread wheat, the *Ph1* locus is a well-characterized regulator of this process, whose main role is allowing only homologous chromosomes (belonging to the same genome) to pair and recombine during meiosis [57, 94, 103]. In this regard, there are mutant lines that harbor an alternative allele for this locus, for instance *ph1*, which is not functional, thus allowing homoeologous chromosomes to pair and recombine [132]. These homoeologous chromosomes include the ones from wheat, but also chromosomes from species from the secondary and tertiary gene pools of the cereal, during the process of gene introgression, being this a powerfull mechanism for this approach [132]. Since other genes appear to contribuite on this mechanism, other studies are being carried out to better elucidate it.

### Speed breeding

Crop breeding is, or, has been, a process which requires considerable time, usualy several years - as for wheat - until a new improved cultivar can be released. The current increasing demand for food added to a number of other factors, such as the ongoing climate change, put pressure on breeding to accelerate the process. Growing segregating lines out of season, at different locations, and the double haploid method have contribuited in this regard, but *speed breeding* has come as a game-changer to accelerate the plant improvement. It is a very recent approach which ultimately aims to shorten plant’s generation time, accelerating breeding and research programmes, in which wheat has been protagonist, among few other crops [123]. It is basically based on photoperiod, light and temperature manipulation (artificially), in growth chambers and glasshouses, and allows one to achieve up to six generations per year - from seed to seed, for spring wheat [36, 123]. The method not only allows for generation advancing, but also for faster phenotyping for numerous traits, such as flowering time, plant height and disease resistance in wheat [36].

### High-throughput phenotyping

The use of high-throughput phenotyping, aims to evaluate several traits in a large number of plants over a short period of time. This technique is comprised of several highly optimized and automated steps, and emerged also in an attempt to follow the performance achieved through genotyping towards the increasing demands of breeding [2, 14, 24].

This can be done under controlled conditions, such as in growth chambers or greenhouses, using plant-manipulating robots and photographic cameras with temperature sensors, CO_2_ meters and scales for weighing live plants [30, 92]. At field level, tractor-coupled or self-propelled platforms, drones or even satellite imagery can perform the tasks [19, 41, 55, 112]. After data collection, analysis is also differentiated, requiring specific software, such as for image processing [30, 55].

### Final considerations and future perspectives

Agriculture has the challenge of meeting the increasing demand for food by an ever growing world population, and these days in an adverse scenario of climate change, restricted availability of arable land and water and constant evolution of pathogens, among other obstacles. Moreover, the demand for food goes beyond quantity, as quality is also required, especially regarding nutritional aspects. Bread wheat and plant breeding have a crucial role on this task.

Breeding has been responsible for increasing wheat yields and improving many other traits, such grain quality, resistance to biotic stresses, etc. However, the cereal mean genetic gain has to be doubled in the next few decades, in order to meet its global demand. Thus, efforts in the development and implementation of improved strategies must continuously take place in wheat breeding programs.

Classical breeding, which is largelly based on crosses and phenotypic selection has been the most used plant breeding method around the globe for more than one century and is still the main approach these days, responsible for the release of the largest number of cultivars. This approach will still be applied as the main or even unique strategy for several years to come, specially in developing countries. It will be gradually replaced to a certain extent by improved methods, again firstly in developed countries, next, in developing ones. Crosses may be replaced by direct insertion of a gene of interest through gene editing and phenotypic selection by GS. However, the complete extinction of the classical breeding cannot be even conceived. Instead, combined approaches will probably predominate in breeding programs. Crosses followed by speed breeding practices and high-throughput phenotyping for selection or GS is a simple example of a combined scheme.

Gene editing and GS are the current cutting-edge approaches in plant breeding. Both can still be improved to deliver more effective results, which will probably happen within the next decade. However, the most important “improvement” required from these methods resides on the reduction of their costs, which is especially true for GS, as genotyping is still considerably expensive. As science and technology continue to move towards., it is difficult to even predict which advance will become available for breeders in two or three decades.

Plant breeding has experienced innovations and revolutions throughout its existence and wheat has been witness to most, if not all, of these transformations and probably will continue as an ally of the transformations to come.

## Data Availability

Data sharing is not applicable to this article as no datasets were generated or analysed during the current study.

## Abbreviations

AFLP: Amplified Fragment Length Polymorphism
CIMMYT: *Centro Internacional de Mejoramiento de Maíz Y Trigo* (International Center for Maize and Wheat Improvement)
CRISPR/Cas9: Clustered Regularly Interspaced Short Palindromic Repeats / CRISPR-associated protein 9
DArT: Diversity Arrays Technology
FAO/IAEA: Joint: Food and Agriculture Organization of the United Nations / International Atomic Energy Agency
GM: Genetically modified
GS: Genomic Selection
*Ph1*: *Pairing homoeologous 1* (gene)
*Ppd*: *Photoperiod* (gene)
QTL: Quantitative Trait Loci
RFLP: Restriction Fragment Length Polymorphism
*Rht*: *Reduced height* (gene)
SNP: Single Nucleotide Polymorphism
SSR: Simple Sequence Repeats
TILLING: Targeting Induced Local Lesions IN Genomes
